# *Fusarium proliferatum*-induced chronic lip ulcer: successful treatment with itraconazole: a case report

**DOI:** 10.1186/s13256-022-03575-5

**Published:** 2022-09-30

**Authors:** Fatemeh Mohaghegh, Bahareh Abtahi-Naeini, Elahe Nasri, Parisa Badiee, Hamid Morovati, Hamed Fakhim, Aida Farmani, Mohsen Meidani, Maryam Ranjbar-Mobarake, Rasoul Mohammadi

**Affiliations:** 1 Department of Dermatology, Skin Diseases and Leishmaniasis Research Center, School of Medicine, Isfahan University of Medical Sciences, Isfahan, Iran; 2 Pediatric Dermatology Division of Department of Pediatrics, Imam Hossein Children’s Hospital, Isfahan University of Medical Sciences, Isfahan, Iran; 3 Skin Diseases and Leishmaniasis Research Center, Isfahan University of Medical Sciences, Isfahan, Iran; 4 Infectious Diseases and Tropical Medicine Research Center, Isfahan University of Medical Sciences, Isfahan, Iran; 5 Clinical Microbiology Research Center, Shiraz University of Medical Sciences, Shiraz, Iran; 6 Department of Parasitology and Mycology, School of Medicine, Shiraz University of Medical Sciences, Shiraz, Iran; 7 Department of Dermatology, Isfahan University of Medical Sciences, Isfahan, Iran; 8 Department of Infectious Diseases, Imam Khomeini Hospital Complex, Tehran University of Medical Sciences, Tehran, Iran; 9 Department of Microbiology, North branch Islamic Azad University, Tehran, Iran; 10 Department of Medical Parasitology and Mycology, School of Medicine, Infectious Diseases and Tropical Medicine Research Center, Isfahan University of Medical Sciences, Isfahan, Iran

**Keywords:** *Fusarium proliferatum*, Itraconazole, Immunocompetent, Case report

## Abstract

**Background:**

*Fusarium* species are saprophytic fungi with a worldwide distribution. These fungi cause various infections among immunocompromised patients; however, they can also involve immunocompetent individuals.

**Case presentation:**

We report a case of a 41-year-old Iranian woman who presented with ulcerative lesions on her lips 10 months ago. She had a long history of anxiety but had no history of classical risk factors such as trauma, cosmetic lip tattoo, burning in her lips, smoking or use of alcohol and opium. A skin biopsy from the lower lip was performed and sent for microbiological examinations. Hyaline septate hyphae were seen on direct microscopy with potassium hydroxide. The clinical specimen was subcultured on sabouraud dextrose agar with chloramphenicol and prepared for antifungal susceptibility testing and molecular identification. Considering the minimum inhibitory concentrations (MIC) for antifungals, itraconazole (100 mg orally twice a day) was started for her, and after 2 months, the lesions were treated. She followed up for 3 months, and no signs of disease recurrence were observed.

**Conclusions:**

Selecting an appropriate treatment strategy according to the laboratory assessments is essential in clinical practice and the management of rare infections to prevent related mortality and morbidity of opportunistic fungal infections.

## Introduction

The genus *Fusarium* is one of the neglected saprophytic fungi with a worldwide distribution that is broadly disseminated in soil, organic materials, and plant debris. This group of fungi can cause various diseases such as onychomycosis, keratitis, osteomyelitis, or sinusitis in immunosuppressed patients [1]. Although it is becoming clear that opportunistic infections usually presented in an immunocompromised state, they can also involve immunocompetent individuals. Chae *et al*. reported a case of invasive fungal pneumonia by *Fusarium* in an immunocompetent patient treated with amphotericin B [2]. Localized mucocutaneous lesions due to the *Fusarium* can be a rare presentation of this opportunistic infection [3]; however, Bodey *et al*. [4] described six primary localized skin infections due to *Fusarium* among patients with hematologic malignancies and neutropenia. In this regard, physicians should be aware of the unusual presentation of infection. This report describes a rare case of chronic lip lesion caused by *Fusarium proliferatum* in an immunocompetent patient. Moreover, we had a review of the related literature.

## Case presentation

**Fig. 1:**
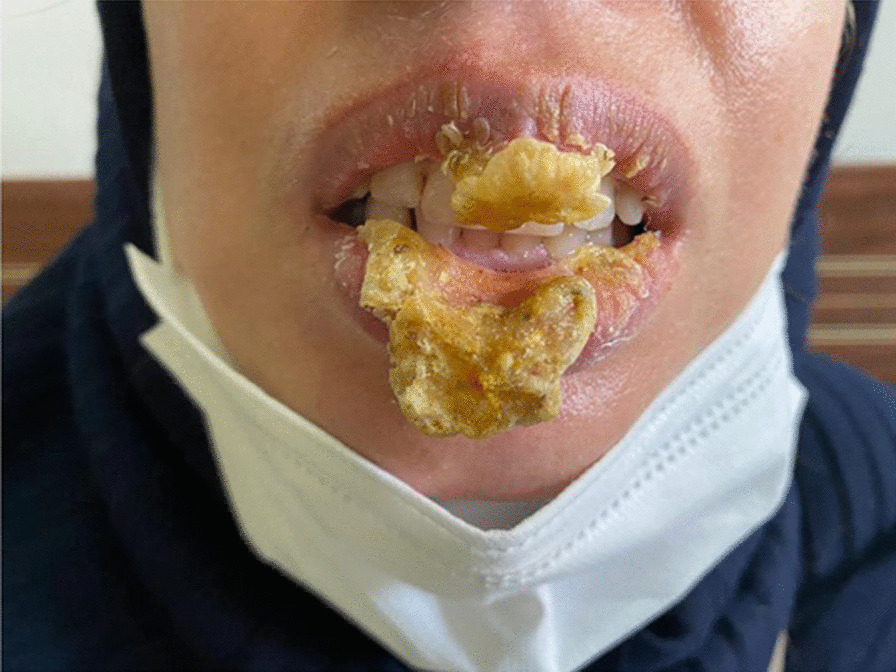
*Fusarium*-induced chronic lip ulcer. Thick crusted ulcerative lesion on the swollen lips

**Fig. 2:**
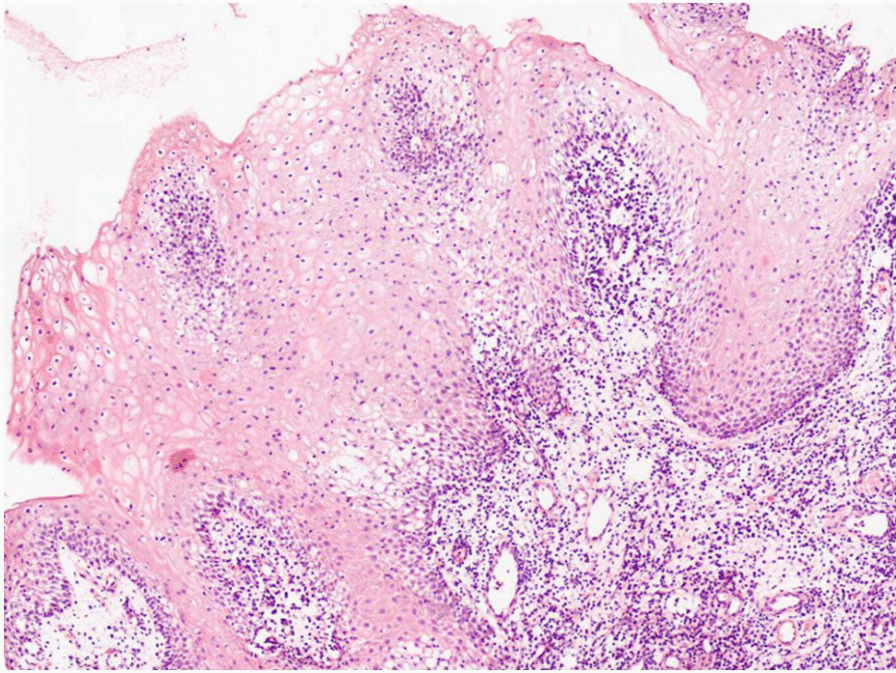
Histopathological feature of lip ulcer. The section showed neutrophilic parakeratosis, acanthosis, spongiosis and exocytosis of inflammatory cells in association with moderate to severe dermal mixed infiltrations. There was no evidence of cleft formation or malignant transformation. (Hematoxylin and eosin stained, ×40)

**Fig. 3:**
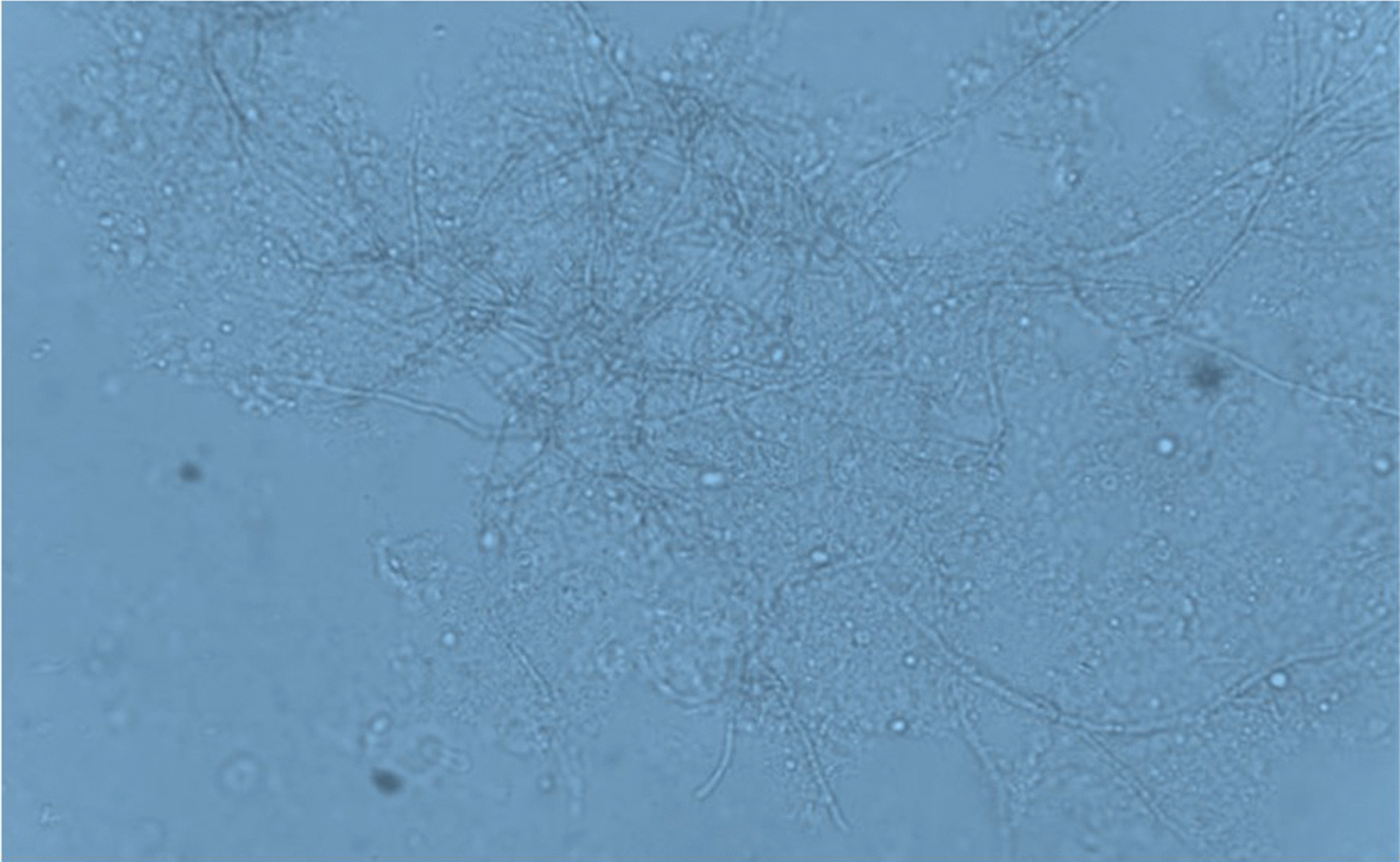
Direct microscopic examination of *Fusarium*. Direct microscopic examination with potassium hydroxide 10% shows hyaline septate hyphae in the crusted lesions of lips

**Fig. 4:**
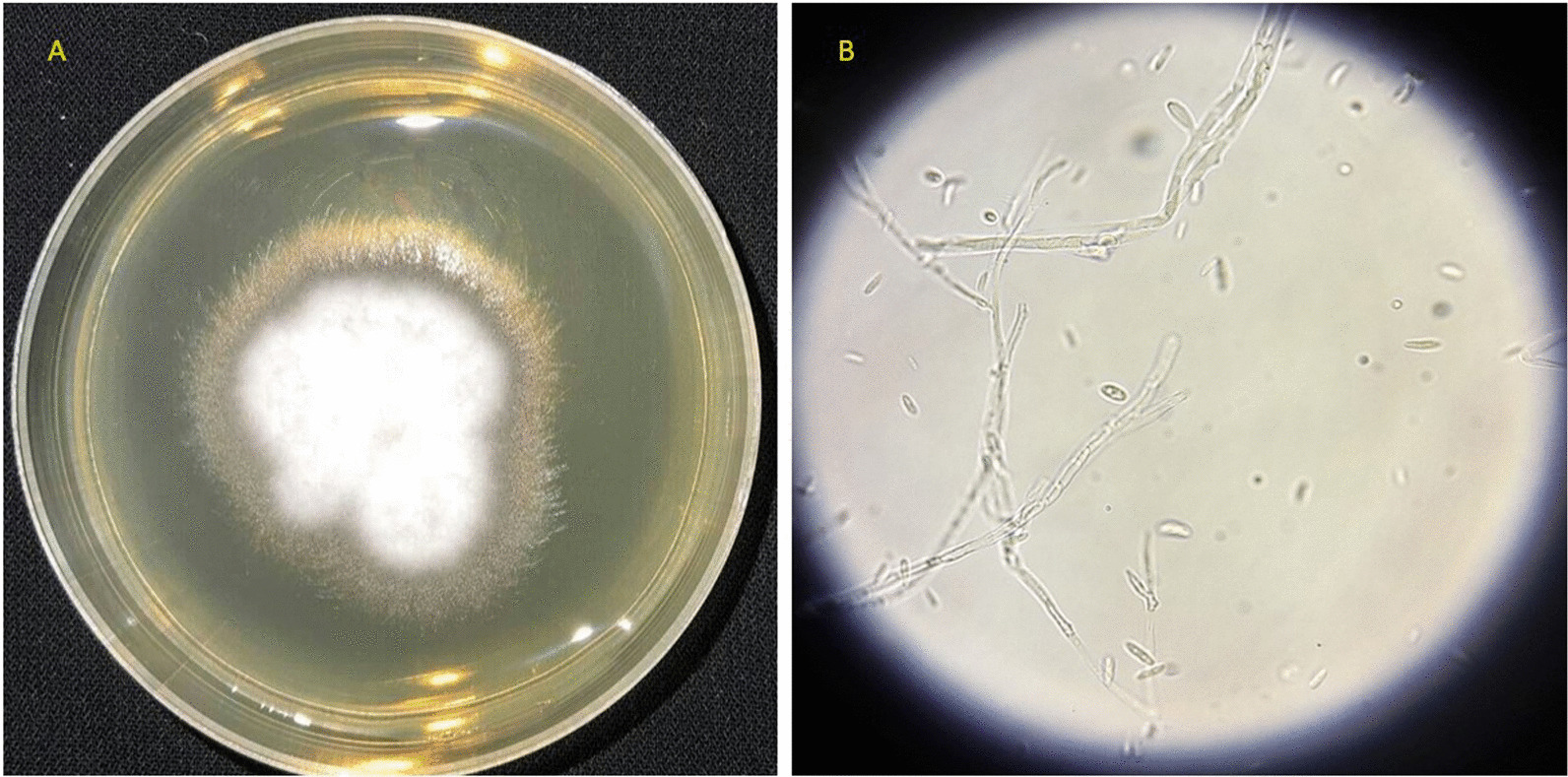
Pink cottony colonies with irregular margin on SDA (**A**), fusiform microconidia and monophialides (**B**)

**Fig. 5:**
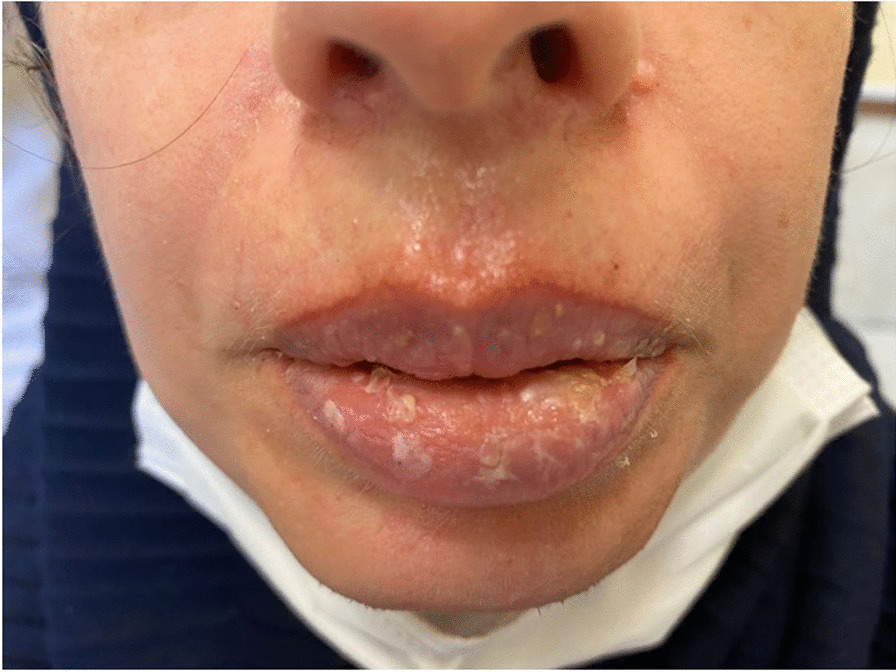
Successful treatment of *Fusarium*-induced lip ulcer. Recuperation of lesions after treatment with itraconazole

**Fig. 6:**
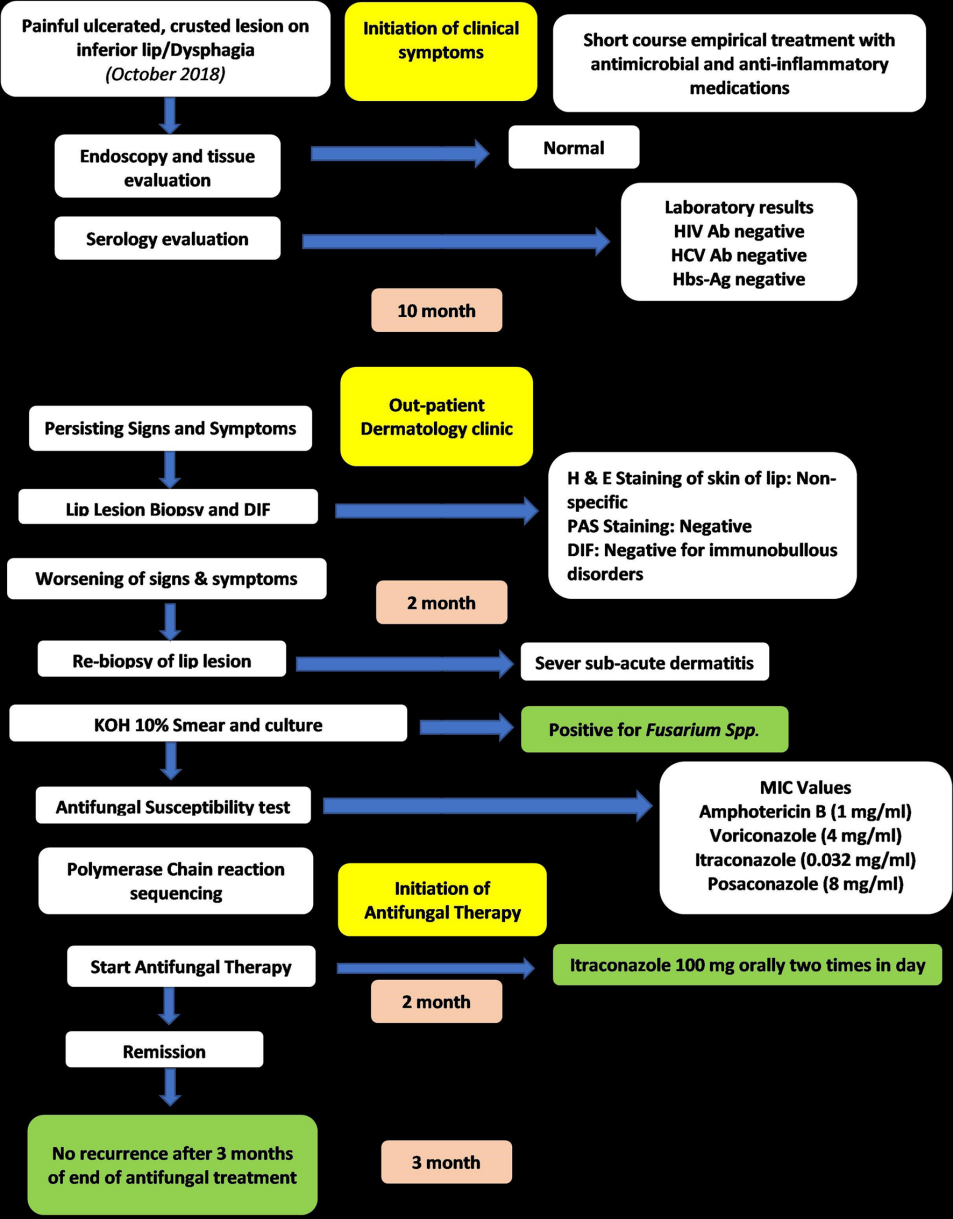
Clinical course timeline. *HIV* Human Immunodeficiency Virus, *Ab* antibody, *HCV* hepatitis C virus, *HBs-Ag* Hepatitis B Virus Surface Antigen, *H&E* hematoxylin and eosin, *PAS* periodic acid–Schiff, *DIF* direct immunofluorescence, *KOH* potassium hydroxide, *MIC* minimum inhibitory concentration

A 41-year-old Iranian woman, without any history of known immunodeficiency conditions presented with slowly growing crusted, painful, and ulcerative lesions on her lips since 10 months ago. The patient had no history of smoking, alcohol, and opium use. She had a history of anxiety symptoms with no specific diagnosis. There was no travel history or unusual behavior. The patient had no classical risk factors for the development of opportunistic infection, prominent history of trauma, cosmetic lip tattoo, or burning in her lips. She had taken corticosteroid nasal spray for bronchitis. The clinical examination revealed an ill-demarcated, kissing ulcer associated thick, crusted area with a size of 3 × 1 cm on her lower lip. The ulcer was tender and discharged when pressed (Fig. 1). There were no symptoms of interdigital intertrigo involvement nor signs of onychomycosis. The patient was not febrile, and there was no regional lymphadenopathy. The oral mucosa and tongue tissues were normal. Systemic general examinations were not noticeable. The results of screening for human immunodeficiency virus antibody (HIV Ab), hepatitis C virus (HCV) Ab, and hepatitis B virus surface antigen (HBsAg) were negative. At the onset of symptoms, she complained of dysphagia; however, she had typical results for endoscopic evaluation. Despite the local wound care and topical antifungal and antimicrobial therapy, immunosuppressive treatment with suspicion of immunobullous disorders and long-term antibiotic therapy worsened the wound. The clinical differential diagnosis of squamous cell carcinoma, other malignant lesions, ulcerative leishmaniasis, ulcerative mycobacterial infection, erosive lichen planus, immunobullous disorders, and opportunistic fungal infection was considered. Following these clinical manifestations, incisional skin biopsy samples were collected from the lower lip twice. Histopathological examination with hematoxylin and eosin (H&E) staining revealed parakeratotic hyperkeratosis, severe acanthosis, spongiosis, and exocytosis of inflammatory cells in the epidermis accompanied by edema in papillary dermis with several inflammatory cells around blood vessels which were nonspecific (Fig. 2). Direct immunofluorescence evaluation was negative for immunobullous diseases. The direct smear for leishmaniasis was negative. Nitroblue tetrazolium (NBT) test was negative. On direct microscopic examination (DME) with potassium hydroxide (KOH 10%), hyaline septate hyphae were seen (Fig. 3). After 6 days, pink cottony growth with an irregular margin was grown on sabouraud dextrose agar (SDA) (Merck) with chloramphenicol (Fig. 4A), and fusiform microconidia and monophialides were seen in the microscopic scene (Fig. 4B). Antifungal susceptibility testing was considered using broth microdilution according to the clinical and laboratory standard institute methods [5]. The MIC values were as follows: amphotericin B (1 mg/mL), voriconazole (4 mg/mL), itraconazole (0.032 mg/mL), and posaconazole (8 mg/mL). For molecular identification, the ITS1‑5.8SrDNA‑ITS2 region was amplified using ITS1 (5′-TCC GTA GGT GAA CCT GCG G-3′) and ITS4 (5′-TCC TCC GCT TAT TGA TAT GC-3′) primers [6]. The PCR products was sent to sequence analysis in a forward direction (Bioneer, South Korea). The results were analyzed with Chromas 2.4 software (Nucleics Pty Ltd, Sydney) and the NCBI BLAST online tool targeting fungal sequences in DNA databases (Nucleotide Blast online tool). *Fusarium proliferatum* was identified as the etiologic agent (having 100% sequence identity), and the gene sequence was deposited in the GenBank with the accession number OK340646. Based on the results of antifungal susceptibility, itraconazole (100 mg orally twice a day) was started, and after 2 months, the lesions were treated (Fig. 5). After a 3-month follow-up, there was no sign of recurrence. Figure 6 shows the clinical course timeline of the present case.

## Discussion

This study presents the first case of *Fusarium proliferatum*-induced lip ulcer in an immunocompetent state which was an unusual manifestation of a rare infection. Another point that makes our case very impressive was its susceptibility to itraconazole and subsequent successful treatment without recurrence of infection with oral itraconazole. Sometimes chronic mucocutaneous ulceration represents an underlying clinical problem and diagnostic challenge affecting the quality of life. Opportunistic fungal infections of the skin are becoming more prevalent with the increased use of single and multi-agent immunosuppressive medications [7]. Known risk factors for these infections include severe illness and debility, hematopoietic malignancy, diabetes mellitus, hematopoietic or solid-organ transplantation, long term or massive doses of antibiotics, long-term of parenteral nutrition, drug addiction, human immunodeficiency virus (HIV) and use of immunosuppressants [3]. The clinical manifestations of *Fusarium* infections depend on the route of infection and the host immune system condition. The genus *Fusarium* is an opportunistic fungus with a worldwide distribution that has been associated with various infections such as keratitis, fungus ball, paranasal sinusitis, and abscesses caused by plant thorns [8]. The most common mucocutaneous manifestations of the infection are umbilicated or necrotic papules, pustules, abscesses, cellulitis, and subcutaneous nodules. While in immunocompetent hosts, onychomycosis and keratitis are the most common forms [9]. Localized skin lesions in immunosuppressed patients should be considered as they may be a sign of disseminated and life-threatening fusariosis. Nevertheless, little is known about *Fusarium* cutaneous infections in healthy people. In immunocompetent conditions, cutaneous infections by *Fusarium* are characterized by preceding skin breakdown, localized involvement, slow pace of progression, and acceptable response to therapy [9]. In localized deep fungal mucocutaneous infections, especially in immunocompetent patients, a history of direct trauma is an important clue for infection. The patient we reported here had no well‐defined and classical risk factors for opportunistic infection. Also, she had no remarkable history of trauma, cosmetic tattoo, or burning on her lips. The diagnosis of *Fusarium* infection is established by detecting fungal elements in tissue. This can be performed by conventional microbiological methods or molecular techniques. In the present case, the diagnosis of *Fusarium* species was carried out by direct visualization of fungal hyaline hyphae in scrapings and positive culture, leading to administration of the antifungal susceptibility testing and molecular identification. Since the microscopic and macroscopic features of the *Fusarium* species are frequently variable in subculture scenes [10], species identification becomes challenging by traditional tests. However, in the majority of clinical cases, the identification performed by phenotypic methods [11], the species remains unidentified. The ITS sequence was applied to identify the current isolate. Although elongation factor (*TEF1*) has been widely used for species identification in the genus *Fusarium*, application of the nuclear ribosomal ITS sequence should be worthy of placing most strains within the relevant complex. This region will not be able to discriminate among individual species within the complex [12] (one of the limitations of the present study). Still, it is reliable for confirming the phenotypic diagnosis. Patients with localized infection usually need surgical debridement and topical antifungal drugs [3]. The routine antifungal susceptibility pattern of *Fusarium* species reveals the resistance to most antifungals; however, various species may have different susceptibility patterns [13]. In general, *Fusarium* isolates exhibit quite high MIC values for ketoconazole, flucytosine, fluconazole, miconazole, posaconazole, and itraconazole, and low MICs of natamycin, econazole and amphotericin B [14]. In contrast to the report of Nucci *et al*. [3] that all *Fusarium* strains were resistant to itraconazole, the current isolate was susceptible to itraconazole (MIC: 0.032 mg/mL). Some experts have suggested combination therapy with the justification that since most *Fusarium* species exhibit high MICs for voriconazole, it would be trusty to start treatment with amphotericin B and an azole (generally voriconazole) [15]. *Fusarium* species show a large variability of resistance to antifungals. For example, *F. dimerum* is commonly susceptible to itraconazole and voriconazole, whereas *F. oxysporum* is more susceptible to terbinafine than other antifungal agents [16]. All *Fusarium* species are resistant to fluconazole; nevertheless, susceptibility to posaconazole is variable [17, 18]. This is an important point in clinical practice when selecting empiric antifungal therapy in patients with clinical suspicion of localized opportunistic fungal infections and unusual presentation; uncommon species also should be considered for initiating antifungal therapy.

## Conclusion

In summary, opportunistic mycotic infection associated with ulcerative lesion should always be considered in any localized chronic mucocutaneous ulcer in both immunocompromised and immunocompetent individuals. This is a rare case of treating *Fusarium* infection with itraconazole. Although resistance to itraconazole in *Fusarium* species have been reported in most studies, the results of the present study suggest the necessity of drug susceptibility testing of clinical isolates in specialized laboratories to use the best treatment strategy in the clinic.

## Data Availability

All data generated or analyzed during this study are included in this published article.
